# End-user perspectives on the development of an online intervention for parents of children on the autism spectrum

**DOI:** 10.1177/1362361320984895

**Published:** 2021-01-11

**Authors:** Susannah Hermaszewska, Jacqueline Sin

**Affiliations:** 1 University of Reading, UK; 2 City, University of London, UK; 3 St George’s, University of London, UK

**Keywords:** autism, eHealth, family caregiver, online intervention, parents

## Abstract

Strong evidence suggests that parents of children on the autism spectrum face increased stressors to their physical and mental wellbeing due to the demands of caregiving and paucity of support. The disparity between the services currently offered and unmet needs indicates that accessible and flexible support through online delivery is needed for parents. This study presents the ideas and perspectives collected from five focus groups of 17 parents of autistic children, on the optimal content and implementation strategy of an online intervention. Using thematic framework analysis, we derived themes under the categories: the need for online interventions; content and design; and implementation. Parents supported the development of a multicomponent online intervention which combines psychoeducation with peer-support and access to healthcare professionals.

**Lay abstract:**

Parent caregivers play an essential role in the lives of individuals on the autism spectrum. The demands of caregiving can have negative effects on the mental and physical wellbeing of parents. Different types of formal support have been developed to help parents to cope with caregiving; however, many parents struggle to access services due to limited availability and busy schedules. The Internet could offer parents more accessible and flexible support. We asked 17 parents what content they would like to include in an online resource. Parents told us about their experiences trying to access and use existing formal support and websites. They overwhelmingly supported the development of an online resource informed by their suggestions. Parents emphasised the need for easier access to information through educational components and direct access to healthcare professionals online. Parents also wanted help with finding existing services and reliable, locally relevant information. Parents stressed the need for a safe environment to meet and chat with other parents online. This research forms the first stage in the development process of an online health resource for parents.

Parenting children on the autism spectrum is associated with greater parenting stress relative to parenting typically developing children and children with other developmental disabilities (Hayes & Watson, 2013). Child characteristics such as low adaptive functioning (Hall & Graff, 2011), comorbid mental and physical health conditions (Valicenti-McDermott et al, 2015) and increased levels of problem behaviours, such as aggression and hyper-activity in autistic children (Estes et al., 2009), are positively associated with increased parent stress. Increased demands of caregiving are often compounded by the experience of lower average household income (Montes & Halterman, 2008), more hours spent caregiving per day (Tunali & Power, 2002) and more frequent and intense marital problems (Hartley et al., 2010) for parents of autistic children in comparison to parents of typically developing children. Supporting parents to manage these sources of stress and enhancing parent capacity to cope are important ways to improve parent psychological well-being.

The relationship between stressors and parental capacity to cope can be explained by the stress-appraisal-coping theory (Lazarus & Folkman, 1984). This theory proposes that the impact of stressors on an individual is determined by the resources that are accessible to support coping. Capacity to cope is enhanced through the use of internal resources (e.g. family communication and cognitive appraisal strategies) and support mechanisms external to family life (e.g. social support and formal support). Coping strategies and interventions work to either lower the actual distress (i.e. emotion-focused coping) or manage the source of distress (i.e. problem-focused coping).

Internal and external resources have been found to mediate the effect of stressors, such as problem behaviours, on mental health outcomes of parents of autistic children (Dunn et al., 2001; Hastings & Brown, 2002). Parents, who have advantageous internal resources and access to strong support systems, have better coping capacity. On the contrary, lack of skills, knowledge and support for parents often results in higher levels of stress in families with autistic children.

Conventionally, interventions such as peer-support, psychoeducation, self-management and family therapy, are delivered face-to-face and designed to increase coping capacity for parents of autistic children (Da Paz & Wallander, 2017). These interventions have had limited success due to substantial barriers in implementation. Barriers include lack of funding or resources causing limited service provision (Galpin et al., 2018; Wodehouse & McGill, 2009); inability to reach families hardest to engage with, for example, who live rurally or from an ethnic-minority background (Fox et al., 2017; Thomas et al., 2007); a historic neglect of research into interventions to support carers (Sin et al., 2018); and subsequent lack of specialised care providers in geographically dispersed areas (McGill et al., 2006). Where interventions have overcome such barriers, parents often do not have the flexibility and time to attend regular meetings or take part in studies assessing intervention effectiveness (Arksey et al., 2003; Bonis, 2016; McGill et al., 2006; Spain et al., 2017). These experiences point to a need to re-evaluate the current delivery mode of interventions in order to reach more families, reduce service costs and make specialist services available to a heterogeneous and geographically dispersed population. Most carers do not need high intensity therapy, instead there is a desire for easier access to information, connections with peers and strategies for coping, all of which are feasible to deliver online (Sin et al., 2018).

Online resources for parents do exist and in one survey 86% of parents, immediately post-diagnosis, sought information about autism spectrum disorder (ASD) on the Internet (Mackintosh et al., 2006). The National Autistic Society, a leading UK charity, receives tens of thousands of unique visitors to their website each month (Stephenson et al., 2012). However, parents report ambivalence towards information sourced online, citing lack of credibility and untailored information as reasons why they might avoid information from the Internet (Gibson et al., 2017). Indeed, a review of 20 autism-related websites (which included charity, encyclopaedia, government agency and clinic sites), found that only six websites provided research references (Grant et al., 2015). Stephenson et al. (2012) found that advice given by eight national autism websites on 33 educational and therapy interventions was largely inconsistent with scientific evidence.

Online interventions, otherwise known as eHealth or digital interventions, are defined as healthcare delivered to patients and carers using the Internet (Eysenbach, 2001). A recent systematic review found that conventional interventions for carers supporting a loved one with long-term illness have been adapted for delivery online with a high level of acceptability and effectiveness for end-users (Sin et al., 2018). In the field of eHealth, where the autonomy of the individuals using the intervention is emphasised, the term ‘end-users’ is commonly used to refer to the people receiving the care/services of online interventions, for example, parents and family caregivers of the proposed intervention. Complex online interventions which comprise multiple components, such as psychoeducation, self-care and peer-support, are seen as providing a probable cost-efficient alternative (Sin et al., 2018). Complex online interventions are advantageous as they enable users to individualise their usage and content choice suiting their own needs and preferences (Sin et al., 2019; Todd et al., 2013).

Despite the promise of online interventions for carers, a review of existing literature on eHealth interventions identified only five empirical studies which satisfied the following inclusion criteria: (1) intervention delivered online; (2) targeting parents of autistic children; (3) featuring synchronous or asynchronous interaction between the provider and end-users; and (4) aimed at supporting parents’ mental health.

Whitney and Smith (2015) targeted maternal stress and relationships in a weekly online journal writing intervention conducted in the United States. The study showed that emotional disclosure through an online medium can effectively reduce maternal stress. The authors suggested that a combination of psychoeducation and emotional disclosure could be a promising approach for future online interventions.

Clifford and Minnes (2013) evaluated an online peer-support intervention for 45 parents of autistic children living in Canada. The parents determined the frequency and schedule for online group meetings. Asynchronous discussion boards were also available for parents to use outside meeting times. Although psychological health outcomes, such as mood, anxiety and stress, went unchanged, parents were satisfied with the opportunity to connect with peers. Parents commented that intervention effectiveness was negatively impacted by differences in the characteristics of their children and geographical location. The diversity of parents’ needs made it difficult for the intervention to suit everyone.

Hemdi and Daley (2017) recruited 62 participants from Saudi Arabia in a randomised controlled trial of a psychoeducation intervention consisting of an initial face-to-face session with a therapist and four individual 30-min sessions delivered by WhatsApp. The sessions aimed to reduce maternal stress and child behavioural problems through increasing parent knowledge of ASD, understanding of stress and behaviour management. Although the intervention successfully reduced stress and depression levels and enhanced parent wellbeing, additional forms of support were recommended.

Similarly, Zimmerman (2014) reported an online psychoeducation and emotional support intervention delivered to eight parents in the United States. Parents received group therapy in four 90-min sessions which covered stress reduction, individualised education plans, puberty and transition to adulthood. The study found that parent knowledge of the topics increased and levels of stress decreased. Overall, parents enjoyed the ease and convenience of the online intervention, despite some having problems using the technology.

Bekhet (2017) reported on a pilot randomised controlled trial exploring the acceptability and feasibility of online PowerPoint presentations on positive thinking training for 64 parents of autistic children in the United States. The participants had split opinions over the most useful content which suggests that interventions should have tailorable content if they are to meet the diverse needs of parents.

The above studies indicate that online delivery of support for parents of children on the autism spectrum has the potential to overcome access and implementation barriers faced by conventional, face-to-face, intervention delivery. However, this research, from only three countries, has so far failed to involve end-users in intervention design. Coproduction of interventions with the end-users themselves is essential for the development of effective online interventions which utilise parents’ experiential knowledge and prioritise their needs. This study therefore asks: From the end-users’ perspectives, how can online interventions best be designed to meet their needs? This study aimed to explore the ideas and perspectives of parents of children on the autism spectrum on the optimal content, design and implementation of an online intervention.

## Method

### Design

This study used a qualitative research method, specifically a focus group design, with 17 parents of children on the autism spectrum who volunteered to give their suggestions for the optimal design of an online intervention to address their diverse needs. A focus group design was chosen to maximise the range of perspectives and insights shared by participants (Kitzinger & Barbour, 1999). The stimulation of discussion caused by group interaction added value to the qualitative research process as it interwove consensus views and personal experience and encouraged more reluctant speakers to contribute (Morgan, 1997). Eborall and Morton (2017) highlight that this is salient when exploring the design of an intervention that moves away from conventional means of delivery or uses new technology.

### Sampling and recruitment

This study required participants to be primary caregiver relatives, aged 18 years or above, able to travel to the focus group location and able to converse in English. Convenience sampling and snowballing techniques were used to recruit participants from schools, voluntary organisations and a clinic in London and South East England. The use of multiple recruitment channels meant that participants were recruited from diverse socio-economic backgrounds. Interested participants were sent the study information sheet and offered the opportunity to ask any questions. Eligible participants were invited to meetings which were held at a time and venue that was most convenient for the majority of participants.

### Study procedure

Two or three members of the research team attended each of the five focus groups and facilitated the discussion. Each participant gave their informed, written consent and was offered £20 as travel expense reimbursement and goodwill payment. The groups started with a warm-up exercise sorting post-it notes with suggestions for the online intervention content such as information on particular topics, and peer-support. A semi-structured topic guide with domains established from previous research into online interventions for carers was used (Lobban et al., 2011; Sin, 2013; Todd et al., 2013). Questions asked to the parents included: What are the parents’ personal experiences of information seeking and peer-support? What are their views on the core content that the resource needs to cover? What are their preferred formats for resource content? What support would parents need to enhance their use of the resource? The meetings lasted on average 110 min and took place in a university facility or a residence in South West London. The focus groups were concluded with a verbal summary of the discussion and researcher insights to check for accuracy of understanding with the participants.

All procedures performed in the study were in accordance with the 1964 Helsinki declaration and its later amendments. The Research Ethics Committee of School of Psychology & Clinical Language Sciences, University of Reading gave ethical approval for this study on 8 May 2018.

### Qualitative analysis

The first author (S.H.) transcribed the audio files verbatim, checked them for accuracy and anonymised participant identifying information. The data were analysed in four phases using thematic framework analysis (Ritchie et al., 2014), with the software Nvivo 12 (QSR International Pty Ltd, 2018).

In the first phase of the analysis, the authors familiarised the data through transcribing the audio files, re-reading data and noting interesting aspects. In the second phase, one author (S.H.) coded all the data, a second author (J.S.) coded 20% of the data independently. Flexible codes (phrases which summarised the content of a section of text) were applied across the data set to draw together relevant and interesting data. The codes were reviewed and refined independently through several iterations. In the third phase, initial themes and subthemes reflecting salient patterns in the data were formed by grouping codes together. These were compared and contrasted with data from existing research on the development of online interventions for carers (Gibson et al., 2017; Lobban et al., 2011; Todd et al., 2013). In the fourth phase, the authors cross-referenced, discussed and clearly defined the themes and subthemes over several meetings. Iterative analysis of the transcripts showed that saturation of data was achieved as the final focus group transcript produced no new themes or subthemes (Guest et al., 1995).

This study was coproduced with a parent-researcher from the autism community who also participated in the research.

## Results

### Participant and family demographic information

Five focus groups, each comprising of 3–5 participants, with 17 parents (age: *M* *=* 43.3, *SD* = 5.2, 15 females) were conducted, in total. Nine of the parents had participated in a psychoeducation course soon after their child’s diagnosis. All parents had used UK National Health Service (NHS) and non-governmental autism websites. Employment status but not educational attainment of the participants was recorded. Parents gave non-identifiable details about their children. One of the 20 children (age: *M* *=* 9.4, *SD* = 4.3) was female. The children were diagnosed with a range of ASD traits and comorbidities and the number of years since diagnosis ranged from 0.2 to 8. See Table 1 for participant and children demographic information.

**Table 1.** Participant and children demographic information.

Participant ID	Age	Ethnicity	Bilingual	Employment	Age of autistic children	Diagnoses of children as given by parents	No. of years since diagnosis	Schooling of autistic children
FG1FP1	49	White American	N	Unemployed	7	High-functioning ASD and ADHD	1	Mainstream
FG1FP2	41	White British	N	Full-time	8	ASD and ADHD	4	SEN school
FG1FP3	44	White British	N	Part-time	6	ASD	1	Mainstream
FG1MP1	52	White British	Y	Full-time	11	ASD and minimally verbal	6.3	SEN school
FG1MP2	44	White British	N	Full-time	11	ASD (undiagnosed) and minimally verbal	8	SEN school
FG2FP1	46	N/A	Y	Part-time	6	ASD and minimally verbal	3	Mainstream
FG2FP2	42	European White	Y	Part-time + student	10,8	ASD	7,2	Mainstream
FG2FP3	38	Indian	Y	Part-time	5.5	ASD and social communication disorder	2	Mainstream
FG3FP1	39	White British	Y	Part-time	10	ASD, ADD, vitiligo, and anxiety	6	Mainstream
FG3FP2	41	White	Y	Part-time	7	ASD and expressive language disorder	2.5	Mainstream
FG3FP3	38	White British	N	Homemaker	10,7,5	ASD, ADHD, and dyslexia	2,2,0.2	Mainstream
FG4FP1	51	Irish	N	Part-time	24	ASD, dyslexia, and dyscalculia	7	Not in education
FG4FP2	37	White British	N	Full-time + student	11	ASD, dyslexia, gut transit disorder, and dyspraxia	1	Mainstream
FG5FP1	36	White British	N	Full-time	6	Severe ASD + non-verbal	3.5	SEN school
FG5FP2	47	White British	N	Part-time	13	ASD	0.4	Home-schooled
FG5FP3	41	White British	N	Part-time	9	Asperger syndrome	0.5	Mainstream
FG5FP4	50	White British	N	Mother at home	14	High-functioning ASD	7	SEN school

ADD: Attention Deficit Disorder; ASD: autism spectrum disorder; ADHD: attention-deficit hyperactivity disorder; SEN: special educational needs.

### Main findings and themes

**Figure 1.**
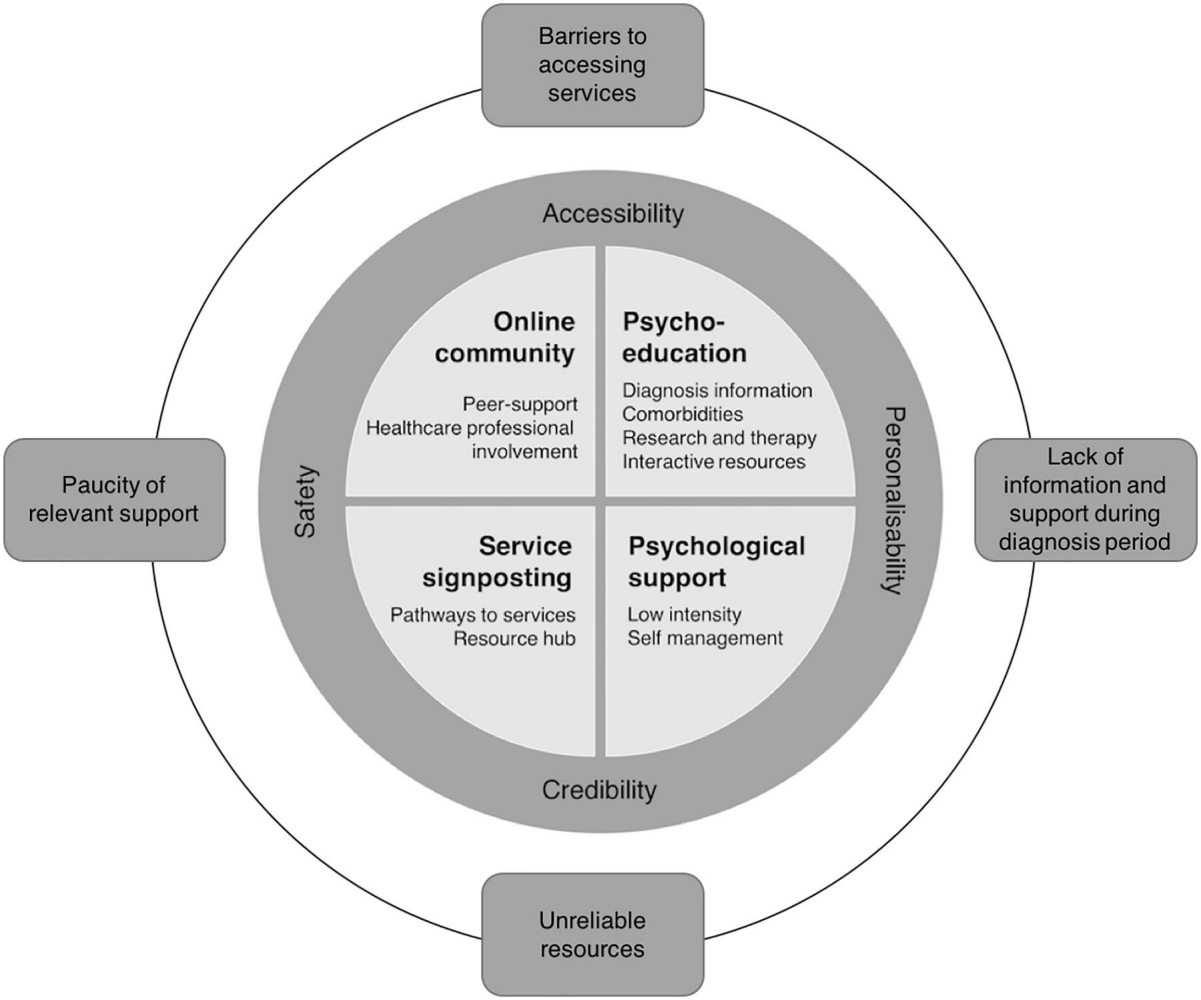
A theoretical model showing the themes derived from the data organised into components of an online intervention.

Themes derived from the data are organised into the following three categories: (1) the need for online interventions; (2) content and design; and (3) implementation. Theme titles can be found in Figure 1.

#### The need for online interventions

The problems parents encountered with formal services (e.g. referral difficulties, waiting times and inadequately trained staff) prevented many parents from accessing local services or even knowing services existed. All parents felt that the support available during the diagnosis period specifically was inadequate and more easily accessible support would have helped themselves, and family and friends, to cope better. Eighty-eight percent of parents resorted to use private healthcare after experiencing problems with the NHS. While parents reported using numerous online resources (at least 14 websites and 10 distinct discussion forums or chat groups), they reported difficulties in finding relevant information, navigating websites and judging credibility. On the subject of searching for support and treatment, one parent said, ‘As a parent you desperately want to help your child, you desperately want a magic wand . . . and so you are actually vulnerable to try things that are not good for your child’. Like this parent, others reported that the paucity of support led to feelings of desperation, vulnerability, isolation and helplessness. All parents agreed that there is a need for the development of an online intervention to respond to the problems with existing services and the impact this has on the psychological wellbeing of parents. The ideas parents gave for the content and design of an online intervention were directly informed by years of problems using formal services.

#### Content and design

Four themes were identified for the content and design of the online intervention: (1) online community; (2) psychoeducation; (3) service signposting; and (4) psychological support.

##### Online community

All participants agreed that an online community would be a valuable way of increasing social and professional support available to parents by facilitating discussion and sharing of events, and resources between parents, healthcare professionals and other members of the community. Essential to the online community was sharing both experiential knowledge and emotional support between parents as peers. As one parent shared, ‘You can find useful bits of information but you might only do that by chatting to somebody and that’s why networking is very good because you get to share what’s worked’. Parents wanted searchable discussion boards with the facility to filter results (by topic, geographical area and condition of child) to enhance the relevance of support. Discussion board topics included: common problems and challenges, local area services and schools, sleep problems, bullying, day trips, crisis support, schools, medication and behavioural traits of ASD.

Parents agreed that incorporating healthcare professionals into the online community in a formal way would be highly beneficial to users because, as one parent contributed, ‘Parents are really craving access to expert advice that is just not available. So yes, I think that that would be really good’. Parents desired a panel of healthcare professionals, ‘that have got like to be a speech and language therapist that has access to it, maybe a dietician, OT, counsellor, psychologist’ to provide expert opinion. Parents discussed using online appointment slots, hour long live drop-ins, post and reply boards, frequently asked questions and webinars as possible ways to increase parent access to healthcare professionals through an online community. Over time, it was hoped that contributions from clinicians will form ‘a knowledge database’ which could be used flexibly by the online community to search for expert advice and information.

##### Psychoeducation

All parents supported a psychoeducational component to deliver information to help them provide more effective care and make informed choices. One parent commented, ‘I think that when somebody has that level of information, then they can go well maybe we do have that problem, I think maybe we would benefit from that’. The participants demonstrated confusion about Diagnostic and Statistical Manual of Mental Disorders (5th ed.; DSM-5) diagnostic criteria for ASD, in addition to behavioural characteristics associated with ASD versus those associated with comorbidities and developmental stages. Parents suggested having an educational component within the intervention to cover these topics. Participants agreed that a pre-ASD-diagnosis section would be particularly helpful, as one parent shared, ‘I was always looking for things like “what are the signs of autism” and things like that . . . so anything like that would be very useful because there’s not anything’.

Thirty-five percent of parents said that information about common comorbidities was essential. One parent recounted her experience trying to support her child’s comorbidity,

He’s been diagnosed with separation anxiety and he actually has a hell of a lot of anxiety around the school, it’s never been addressed . . . it’s almost like the mental health of our children is more important and getting the help for that bit, because the diagnosis we can’t change.

An extensive list of comorbidities was discussed, indicating that the support needs of the parents are diverse. The comorbidities included: gut problems, anxiety, obsessive compulsive disorder, chronic vomiting and diarrhoea, incontinence, attention-deficit hyperactivity disorder (ADHD), allergies, sleep problems, hearing problems, mental health problems including suicidal ideations and learning difficulties.

Parents wanted easier access to the latest updates in scientific research and news regarding autism. One parent stated, ‘I don’t know anywhere, I don’t know any English website with that . . . not really in the research’. Fifty-three percent of participants did not know which, if any, interventions their children received at school and had not integrated therapies at home. One parent commented, ‘You are with your kid all the time so what I was looking for at that time was things I could do with my child at home’. One parent was supported by the other participants in her suggestion to ‘collate like a list of therapies of what parents have tried and what they have found most helpful . . . in a one stop repository’. Therapy and treatment guides (e.g. dietary and feeding, speech and language, occupational therapy and medication) were supported tools to help parents make informed choices about interventions. Parents supported the inclusion of evaluation based on scientific evidence and parent experience of interventions.

All parents supported suggestions for interactive, downloadable and printable resources for psychoeducation, parent-mediated intervention, communication and play. One parent suggested, ‘Films or games or things to improve their vocabulary . . . something that you can do at home. Or routines that you can do to help them’. Parents requested resources for teachers and children. They also asked for personalised resources such as social stories and scaling cards. One parent suggested that content could be recommended to users based on information given in user profiles such as their child’s age, ASD traits or comorbid conditions.

##### Service signposting

Seventy percent of parents requested the inclusion of information on diagnosis pathways and existing local services and online information linked from other websites. One parent captured the sentiment in the room when she said, ‘I need that sign posting as a parent’. Participants expressed interest in having specific information on local diagnosis pathways, how to access local services and alternatives to publicly funded health services. One parent explained why current services are not sufficient,

Navigating through the whole, the whole process from, sort of thinking as a parent there’s something wrong with my child to . . . there’s lots of information out there but it’s all in different little sections you know . . . I just think the whole thing is not integrated.

Parents emphasised the need for more guidance on the support services available while awaiting diagnosis. As two parents said, ‘Actually people are going, waiting a year and a half for this diagnosis thinking something is going to help and it doesn’t’ . . . ‘Yeah, and all that time you could be doing something else for your child. So an online place would be good’. Parents expressed a strong desire for the inclusion of information about service-use assessments and form-filling help to access health, financial and social services, which are available after diagnosis.

Seventy-six percent of parents agreed on the importance of having a central platform or ‘resource hub’ from which users can be signposted to screened, credible online resources and support services. One parent said, ‘Equally important as a whole new resource, is to collate what is already available and make it accessible’. One parent suggested that the hub could be searchable by topic to facilitate easy access to websites such as parent groups, legal information and support, crisis management and support, parent blogs, scientific research developments and training courses.

##### Psychological support

Two parents appreciated that psychological support for themselves could help reduce stress and address other mental health problems. One parent commented, ‘I’m quite sure that’s a good thing’. Others did not support prioritising psychological support for themselves. One parent reasoned, ‘The best thing for my psychological health would be for the schools to give some support to my son’. Importantly, the parents described themselves as feeling isolated, depressed, helpless, desperate, overwhelmed, tired and vulnerable, thus, indicating that they would likely benefit from some form of low intensity psychological support.

#### Implementation

A further two themes were identified relating to logistical concerns for the implementation of the intervention: (1) accessibility and ‘personalisability’ and (2) credibility and safety.

##### Accessibility and ‘personalisability’

Parents emphasised the importance of accessible interventions and the convenience of those delivered online. As one participant said, ‘It’s so easy just to go and pick up your phone or hit the computer’. Parents demonstrated enthusiasm for both asynchronous and synchronised ‘live’ discussion. They desired a blend of video, textual and graphic content, in addition to downloadable content, as an important way to facilitate wider use. Making the intervention available to the wider autism community (e.g. individuals on the autism spectrum, teachers and extended family) and available in different languages was highly desired. The proposed intervention was praised by one participant for having the potential to reach minority groups, ‘In making these [online] resources . . . these other communities, that are not engaging otherwise, therefore get access to things that they are entitled to and resources that they need, it definitely fills a gap’.

The ability to personalise use of the intervention, ‘so you can choose your own approach’, was highly favoured in order to reach a parent population with diverse needs. It was clear that this diversity resulted from having children with diverse spectrum of traits, geographical locations and content medium preferences. Delivery of the intervention through the Internet could provide the flexibility needed to achieve this. As one participant commented,

There are so many kids who are so divergent from each other and they’re all sick and they are all going to this one resource. And they’ll be like, hey this doesn’t work for my child, but it might work for that person’s child.

The parents agreed with one parent who stressed that ‘local information is critical to the day to day stuff’. Parents identified the importance of the intervention being able to cover a wide range of up-to-date information and yet being subject to different users’ needs of individualised tailoring of content and intensity and flexibility of usage.

##### Credibility and safety

Seventy percent of parents supported accreditation of the intervention by the NHS or a university or research institution in order to prove its credibility to end-users. However, 24% of parents demonstrated distrust in the information and services provided by the NHS. One participant stated, ‘I wouldn’t trust the NHS. I would expect it to be really slow and not up-to-date’.

Parents supported having direct access to healthcare professionals in addition to research-based, reliable and safe information. One parent explained this was, ‘so that you can look up all the things you’ve heard on Mumsnet (a generic UK based online forum) and find out whether these things are true’. Parents showed equal support for the experiential knowledge of other parents. Indeed, some viewed the parent community as a preferred source of information and advice over that of professionals and services. For example,

For me, as a parent, I can make my own decision whether I want to try what other parents say because probably in that instance that would have more sense for me what any parents would think about it than any scientist, sorry. That’s a huge difference.

Parents highlighted that it is essential to have registration and agreements to ensure user security and safety. Parents discussed, with some level of ambivalence, the possibility of having private profiles with an option to add details about child and family circumstances, yet in a way that would not identify themselves or their child. The profiles could enable parents to search for relevant information through some kind of automated filtering system. One parent hoped having profiles would facilitate the provision of tailored advice by the healthcare professionals associated with the intervention.

Parents unanimously agreed the online community should be monitored, and one parent added, ‘It’s about who approves what goes in there. Instead of people just posting what they like’. Contributions from users should be screened by experts, as one parent explained, ‘So knowing that there is a reliable body that will check that these things are not harmful in the first place but also helpful’.

### A multicomponent intervention

The themes and subthemes have so far been presented as discrete entities; however, as shown in Figure 1, these themes form a set of interweaving and interacting components of a complex, multicomponent intervention. The problems parents experienced with existing support informed the ideas for the content, design and implementation of the online intervention. For example, negative parent experiences of formal services available pre- and post-diagnosis informed requests for the prioritisation of information about the diagnosis and pathways to assessment and services. The themes and subthemes for content and design are highly interdependent with different aspects of intervention implementation. For example, parents considered the suggestions for an online community as inseparable from ensuring user safety through specific implementation strategies. Parents considered issues of accessibility and ‘personalisability’ to be important to content about available services because only local service information for specific needs is useful. Moreover, the inclusion of existing websites via a resource hub will only work under the wider umbrella of a credible intervention in which the content is professionally screened for quality.

## Discussion

This study aimed to explore the ideas and perspectives of parents of children on the autism spectrum for the optimal content, design and implementation of an online intervention. Parents demonstrated their need for an online intervention through giving examples of the problems they experience with existing services and resources. This included long-waiting times, varying relevance of services, inadequately trained staff and distrust of information credibility. These experiences prompted them to support the idea of an online multicomponent intervention which will allow flexible access and much needed information and support. For the design of an online intervention, parents emphasised the importance of peer-support, direct access to healthcare professionals, credible and relevant information for psychoeducation, easier navigation of existing services and the need for a balance between expert advice and parent opinion. Parents stressed that the intervention must use specific implementation strategies to make it accessible, tailorable and safe for all users.

The results of this study reinforce calls from other family carer groups for multicomponent online interventions, which integrate psychoeducation, peer-support and psychological support, and are delivered to carers in a flexible and accessible manner (Sin et al., 2018). The preference for a multicomponent intervention highlighted in this study might explain the limited effectiveness of previous single-component, non-tailorable, online interventions at improving outcomes for parents of children on the autism spectrum (Clifford & Minnes, 2013; Hemdi & Daley, 2017). Indeed, a recent systematic review identified that a combination of peer-support, professional stress management and problem-solving training and access to reliable information about ASD are essential to effective mental health interventions for parents (Catalano et al., 2018). Existing effective online interventions for family carers more broadly have combined conventional psychoeducation with professional and peer-network support with considerable satisfaction from end-users (Sin et al., 2018). Participants in this study, and in previous research scoping end-user views on the implementation of online interventions, sought interventions which prioritise user safety and flexibly cover a wide variety of topics, while allowing parents to choose content and self-pace use in a manner to fit their individual needs (Sin, 2013; Todd et al., 2013).

The parents emphasised their desire for psychoeducation, delivered online through interactive information components and direct access to healthcare professionals. Two studies have delivered online psychoeducation to promote problem-focused coping in parents of children on the autism spectrum, yet they were limited in their success, possibly by the small range of topics covered (Bekhet, 2017; Hemdi & Daley, 2017). Parents in this study provided a comprehensive set of psychoeducation topics (i.e. in-depth information on ASD-diagnosis, comorbidities, therapy and research) that they felt would be feasible to deliver online. The online delivery of psychoeducation on these topics has the potential to address access problems identified both by the participants in this study and the wider carer population (Wodehouse & McGill, 2009). In addition, a permanent, online ‘knowledge database’ may resolve problems with long-term information retention highlighted by previous studies on face-to-face psychoeducation (Bonis, 2016; McGill et al., 2006). This study indicates that the success of existing online psychoeducation for the wider carer population is likely transferable to parents of children on the autism spectrum (Sin et al., 2018).

A peer-support component was strongly supported by participants, reflecting appreciation of peer-support among parents of children on the autism spectrum (Banach et al., 2010; Galpin et al., 2018; Meadan et al., 2016; Sartore et al., 2013). Some parents had concerns, consistent with views obtained from the wider carer population (Gibson et al., 2017; Sin, 2013), about the safety of online communities. Parents in our sample, therefore, envisioned multiple strategies to ensure user safety. Based on the perspectives shared in this study and evidence of the efficacy of peer-support (Sartore et al., 2013), the development of moderated and accessible online peer-support should be prioritised for this population. Moreover, as preferences for parent expertise versus professional expertise vary within this carer group, an online intervention would be beneficial as it allows parents to choose from whom they source their information.

This study is consistent with previous research in reporting a perceived deficit of expertise in information and support available to parents (Wodehouse & McGill, 2009). Recently developed online interventions for carers have responded to this need through the integration of health and social care experts into discussion forums (Sin et al., 2019). Given that access to individualised expert advice is so difficult to attain, our sample uniformly supported the involvement of healthcare professionals in the intervention. Contrary to parents of autistic children who participated in a study by Gibson et al. (2017), the parents in our sample did not feel that online delivery would inhibit the quality of support that healthcare professionals could provide.

### Implications and further development

Parents perceived the involvement of the community and multidisciplinary agencies as a vital part of their children’s care. Consultation with these key stakeholder groups is needed to complete the theoretical work of the online intervention development. Expansion of the geographical locations of the sample to include more areas of the United Kingdom is needed to investigate if an online intervention would be beneficial to the wider population. Coproducing the intervention, through working collaboratively with parents and other key stakeholders, will be an important step in refining the intervention design before a prototype can be trialled through a pilot study (Craig et al., 2008; Sin et al., 2019; Whittaker et al., 2012). This phase of feasibility evaluation will further explore the relationship between the content and design of the intervention with de facto implementation, potential barriers and desired outcomes.

### Limitations and strengths

One limitation of this study is that several last-minute cancellations by parents meant focus group sizes were smaller than anticipated. We intentionally recruited 4–8 participants for each of the five groups; however, three groups featured three participants which are below the number recommended by Carlsen and Glenton (2011). As data saturation was reached overall, it is valid to conclude that the most commonly held ideas and perspectives of parents were captured. However, the small focus group sizes could have limited the number of atypical views that might have been shared in larger groups with greater participant diversity.

A considerable asset to this study is the involvement of parents, including a parent-researcher, to co-conduct the study. This study placed the end-users at the heart of the intervention conception and development to ensure that their desires, needs and feedback will be woven into every aspect of the intervention development. This study acknowledged the growing demand from the autism community to lead and coproduce the research that affects them (Pellicano et al., 2014).

Reflexivity and dependability were sought through full disclosure of the research process (Mays & Pope, 2000). The strategy taken for this study, including the use of focus groups, coproducing the intervention with its end-users, use of thematic framework analysis, in addition to the concurrent and iterative analysis of data and the collaboration between multiple researchers, has considerably shaped the conclusion reached.

## Conclusion

The results from this study show that parents supported the acceptability and usability of an online intervention for themselves as primary caregivers to their autistic children. This study formed part of the theoretical development phase of designing a new intervention for parents of children on the autism spectrum and the contribution of parents to this process was highly valuable (Craig et al., 2008). Parents had clear ideas about how psychoeducation, peer-support and signposting services should be designed and delivered online to best meet their needs. Parents generated simple solutions to the inundation of fragmented and unreliable information found on the Internet which impedes access to much needed relevant and credible services and information. This study has provided a clear framework, integrating the stress-coping-appraisal theory and expertise from end-users, for an online intervention tailored to the specific needs of parents of children on the autism spectrum.
